# A Review of the LARIAT Suture Delivery Device for Left Atrial Appendage Closure

**Published:** 2015-04-03

**Authors:** Payam Safavi-Naeini, Mehdi Razavi, Mohammad Saeed, Abdi Rasekh, Ali Massumi

**Affiliations:** Department of Cardiology, Texas Heart Institute, Houston, Texas, USA.

**Keywords:** Hear atria, Atrial fibrillation, Equipment and supplies

## Abstract

The prevalence of atrial fibrillation (AF) is 1-2 % in the general population, and the risk of embolic stroke in AF patients is 4-5 times higher than that in the general population. AF-related strokes are often severe, and the rate of permanent disability is much higher among individuals who have AF-related strokes than in those who have strokes unrelated to AF. In patients with AF, more than 90 % of thrombi originate from the left atrial appendage (LAA). The purpose of this paper is to review the efficacy and safety of performing the LAA closure with the LARIAT Suture Delivery Device to prevent AF-related stroke in patients with contraindications to oral anticoagulant therapy.

## Introduction

Atrial fibrillation (AF) is the most common sustained arrhythmia. In the United States, the number of adults affected by AF is currently estimated to be between 2.7 million and 6.1 million, but this number is projected to increase to between 5.6 million and 12.1 million by 2050.^   1 ^ In the general population of North America and Europe, the prevalence of AF is between 1 % and 2 % and increases with age, rising from 0.5 % in individuals 50 to 59 years old to around 9 % in those 80 to 89 years old.^   2^

AF is classified into four different categories on the basis of the presentation and duration of the arrhythmia: paroxysmal AF (terminates spontaneously or with intervention within 7 days of onset); persistent AF (sustained for more than 7 days); long-standing, persistent AF (continuous for more than 1 year); and permanent AF (continuous, and the patient and physician have mutually decided not to pursue restoration and/or maintenance of sinus rhythm).^   3 ^ AF is a major risk factor for stroke, independently increasing the risk of stroke by about 4-5 times.^   4 ^ The risk of AF-related stroke is almost the same for all four types of AF, with an annual rate of 5%; these strokes account for at least 15% of all strokes in the United States.^5^^,^^6^ AF-related strokes are often severe. Therefore, patients who have AF-related strokes are at increased risk of becoming permanently disabled and needing institutional care, and they also have greater short-and long-term mortality rates as compared with patients who have strokes unrelated to AF.^        7 ^

The left atrial appendage (LAA) is derived from the left atrium and forms a trabeculated cul-de-sac with a relatively narrow neck, which creates an appropriate milieu for blood stasis. In patients with AF, more than 90% of thrombi form in the LAA.^        8 ^ Thrombogenesis in patients with AF is a multifactorial process that is caused by endocardial damage, blood stasis, and abnormalities in blood constituents.^   9 ^ Management of AF involves three objectives: rate control, rhythm control, and prevention of embolic stroke. 

AF-related strokes are preventable, and the current recommendations for prevention are as follows: 

1) In patients with a CHA_2_DS_2_-VASc score ≥ 2, oral anticoagulation (OAC) with Warfarin or new alternative antithrombotic agents (e.g. Apixaban, Dabigatran, and Rivaroxaban) is advised.

2) In patients whose CHA_2 _DS_2_-VASc score = 1, Aspirin is optional.

3) In patients whose CHA_2_ DS_2_-VASc score = 0, no treatment (not even Aspirin) is necessary.^   3 ^

Although OAC therapy has markedly decreased the incidence of AF-related strokes, a large percentage of patients with AF (up to 40%) may not receive the appropriate therapy for stroke prevention.^10^^,^^11^ In fact, around 20% of these patients have some degree of intolerance or contraindications to OAC therapy.

For patients with AF who have contraindications to OAC therapy or in whom OAC therapy has failed previously, an alternative method to prevent stroke is to obliterate the LAA. This can be done surgically with instruments such as the AtriClip Device (Atri Cure West Chester, OH, USA) or percutaneously with instruments such as the AMPLATZER Septal Occluder (St. Jude Medical, Plymouth, MN, USA), the PLAATO system (eV3, Plymouth, MN, USA), the WATCHMAN Left Atrial Appendage Closure Device (Atritech, Plymouth, MN, USA), the AMPLATZER Cardiac Plug (St. Jude Medical, Plymouth, MN, USA), or the LARIAT Suture Delivery Device (Sentre HEART Inc., Palo Alto, CA, USA).^   12 ^

In this article, we review the efficacy and safety of using the LARIAT procedure to prevent stroke in patients with AF.

***LARIAT Suture Delivery Device***

**Figure1:**
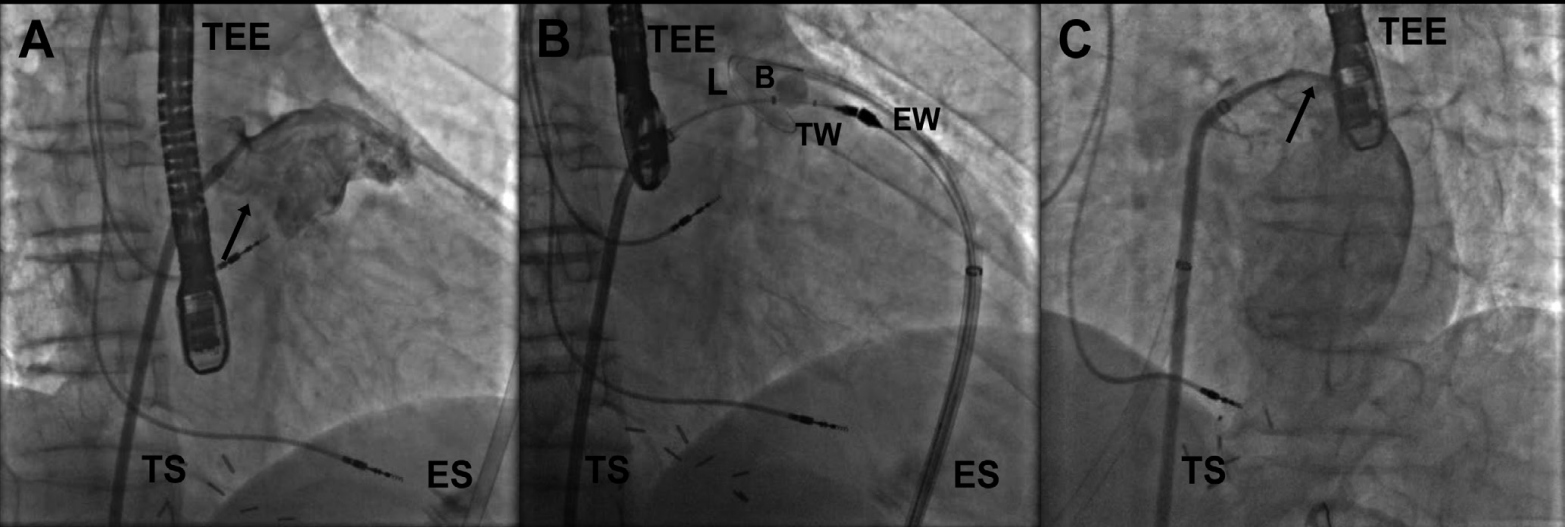
Fluoroscopic images of the LARIAT procedure. A) Delineation of the contours of the left atrial appendage (LAA) by contrast injection through the transseptal sheath placed at the LAA ostium (arrow) in the right anterior oblique view. B) The LAA and epicardial magnet-tipped wires are adjoined. A LARIAT snare (L) is placed at the LAA ostium from within the pericardial space, guided by a contrast-filled balloon. B) placed within the LAA ostium in the right anterior oblique view. C) Left atrial angiogram, showing the exclusion of the LAA in the left anterior oblique view (arrow).

ES, Epicardial sheath; EW, Magnet-tipped epicardial wire; TEE, Transesophageal echocardiographic probe; TS, Transseptal sheath; TW, Magnet-tipped transseptal wire.

Reprinted from Massumi et al.: Initial experience with a novel percutaneous left atrial appendage exclusion device in patients with atrial fibrillation, increased stroke risk, and contraindications to anticoagulation. Am J Cardiol, 2013;111:869-873, with permission from Elsevier.^13^

**Figure 2:**
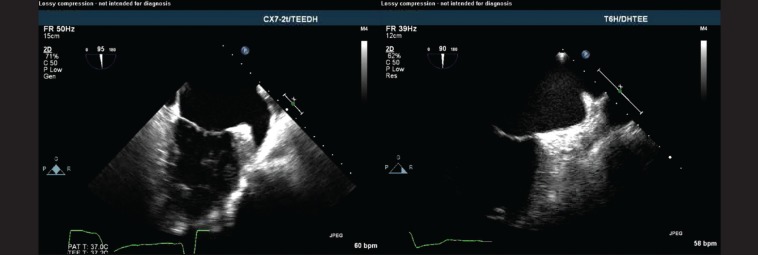
Transesophageal echocardiogram, showing the complete exclusion of the left atrial appendage (LAA). A) Mid esophageal two-chamber view, showing the LAA (arrow) before exclusion. B) Mid esophageal two-chamber view, showing the smooth wall of the left atrium (arrow) immediately after the LAA exclusion with the LARIAT snare

Reprinted from Massumi et al.: Initial experience with a novel percutaneous left atrial appendage exclusion device in patients with atrial fibrillation, increased stroke risk, and contraindications to anticoagulation. Am J Cardiol, 2013;111:869-873, with permission from Elsevier.^13^

The LARIAT Suture Delivery Device, which has 510(k) approval from the United States Food and Drug Administration for soft-tissue occlusion, was invented by Dr. William Cohn, a cardiovascular surgeon at the Texas Heart Institute (THI).The device is used to tie off the LAA, thereby removing the main source of thrombi that cause strokes in *patients with* AF (Figure 1). For those who are appropriate candidates (Table 1),^13^^, ^^14^ the LARIAT procedure is performed by using opposite-pole, magnet-tipped guide wires (FindrWIRZ) with the patient under general anesthesia. The device consists of four main components: 1) a 15-mm–diameter balloon catheter compatible with 9-Fr access (Endo CATH); 2) 0.025-inch and 0.035-inch opposite-pole, magnet-tipped guide wires (FindrWIRZ); 3) a 13-Fr epicardial guide cannula (SofTIP); and 4) a 12-Fr suture delivery device.^        13 ^ One guide wire is placed in the apex of the LAA through a transseptal sheath, and the other is inserted into the epicardial space with the epicardial guide cannula through a subxiphoid puncture and is advanced toward the LAA to bind the other magnet-tipped guide wire. After the two magnetic wires are bound, the LARIAT snare is advanced over the epicardial guide wire and placed on the proximal end of the LAA to tighten a loop stitch around the base of the LAA. After the complete closure of the LAA has been confirmed with Doppler echocardiography (flow < 1 mm on cross section), the suture is released (Figure 2). The suture seals off the LAA from the rest of the heart, and the LAA shrinks afterwards.^        13 ^ The only material that remains in the body is the suture that ligates the LAA, and nothing is exposed on the endocardial side. Therefore, the use of Warfarin or other anticoagulants is not necessary after the procedure, and the patients are usually prescribed Aspirin and Clopidogrel upon hospital discharge. In addition, oral Colchicine is given for 1-2 weeks to prevent postoperative pericarditis. 

**Table 1:** Recommended selection criteria for use of the LARIAT procedure in patients with atrial fibrillation

Inclusion criteria:^ 14 ^
1) CHADS_2_score ≥ 2 or CHA_2_DS_2_-VASc score ≥ 3
2) Contraindications or intolerance to standard OAC therapy (i.e. history of internal or external bleeding or at high risk for bleeding)
3) Failure of OAC therapy (i.e. embolic event despite OAC therapy)
Exclusion criteria:^ 13 ^
1) History of cardiac surgery
2) Myocardial infarction within the previous 3 months
3) Embolic events within the previous 30 days
4) New York Heart Association class IV heart failure symptoms
5) History of thoracic radiation therapy
6) A superiorly oriented LAA or an LAA > 40 mm

OAC, Oral anticoagulation; LAA, Left atrial appendage

***Procedure Outcomes***

The same efficacy and complication rates for the LARIAT procedure were reported for our initial experience at THI^13^ and for studies reported by Bartus et al.^        15 ^ (multi-center study) and Stone et al.^   16 ^ (single-center study) (Table 2). In these studies, the acute success rate of the LARIAT procedure was around 95%. Follow-up transesophageal echocardiography (TEE) showed a 100% LAA closure rate in the THI study^        13 ^ (mean time to follow-up TEE, 96 ± 77 days), a 97 % closure rate in the study by Bartus et al.^        15 ^ (time to follow-up TEE, one-year), and a 100 % closure rate in the study by Stone et al.^   16 ^ (mean time to follow-up TEE, 45 ± 15 days). In contrast, a multi-center study by Price et al.^        17 ^ reported a significantly higher complication rate and a lower acute success rate than the studies cited above. However, the exclusion and inclusion criteria were not defined in the Price study, and follow-up TEE was performed in only about 40% of the patients, a proportion that is substandard based on the data published thus far. Therefore, it would be difficult to analyze the safety of the LARIAT procedure based on the results of that study.^        17 ^ Access-related complications in these studies were primarily associated with pericardial access, but most complications were not major and resolved with medical management. Recently, we performed the LARIAT procedure by using a micropuncture for pericardial access. Based on our clinical experience, we believe that this adjustment could greatly decrease the risk of right ventricular perforation and major pericardial effusion due to the procedure.

## Conclusions

AF-related stroke is a major problem and is sometimes challenging to prevent. Warfarin therapy significantly reduces the risk of stroke. Fewer than 50% of patients who are at risk for AF-related stroke are prescribed Warfarin or fill a prescription for it because of patient preferences or relative or absolute contraindications.         At times when Warfarin should be stopped, for example for surgery or during significant bleeding events, patients with AF are at significant risk of having a thromboembolic event. In addition, randomized clinical trials have shown that only around 60% of serial international normalized ratio (INR) measurements in patients who use Warfarin are within the therapeutic range.^        18 ^ Furthermore, Warfarin use is inconvenient for patients because it requires conitnuous INR monitoring and dose adjustments and because there can be a wide range of drug interactions. Although newer oral anticoagulants overcome many of these difficulties, all anticoagulants increase the risk of bleeding and cannot be used in patients who are at high risk for bleeding events. Because thrombi formed in the LAA cause most thromboembolic events in patients with AF, the LAA closure is the cornerstone of all new methods for preventing AF-related stroke in patients who are not suitable candidates for OAC therapy. The minimally invasive strategies for occluding the LAA ostium include implantation of a foreign body (such as the WATCHMAN Left Atrial Appendage Closure Device) and pericardial suture ligation of the LAA base.

The acceptable acute success rate of the LAA closure with the LARIAT device (> 90%) and the relatively low rate of complications associated with this procedure suggest that LAA ligation with the LARIAT device may be a reasonable option for preventing strokes in patients who have contraindications to OAC therapy. The main difficulty in performing the LARIAT procedure is the need for simultaneous transseptal and pericardial access, which increases the risk of pericardial effusion and bleeding. In addition, the use of the LARIAT device can be limited by anatomic variables such as an LAA diameter > 40 mm, a posteriorly rotated LAA, or pericardial adhesions from prior cardiac surgery or pericarditis. Although the LARIAT procedure has not been shown to be superior to other LAA closure techniques, it could be a desirable choice when there is an absolute contraindication to OAC therapy.

**Table 2:** Published outcomes for the LARIAT procedure: acute success rate, complications, and efficacy for stroke prevention

	Bartus et al.^ 15 ^	Massumi et al.^ 13 ^	Stone et al.^ 16 ^	Price et al.^ 17 ^
Patient population	89	21	27	154
Intent-to-treat	85 (96%)	20 (95%)	25 (93%)	154 (100%)
Procedural success	82 (95%)	19 (95%)	25 (93%)	132 (86%)
Time to follow-up TEE	1 y	96 ± 77 d*	45 ± 15 d*	Not provided
Complete closure in follow-up TEE	64/65 (98%)	17/17 (100%)	22/22 (100%)	50/63 (79%)
CHADS_2_ score	1.9±0.95*	3.2 ± 1.2*	3.5±1.4*	3^†^ (IQR: 2-4)
Patient follow-up for clinical endpoints	1 y	352 ± 143 d* (range: 50-600 d)	4.0 ± 3.4 mo* (range: 0.1-12.7 mo)	112 d† (IQR: 50-270 d)
Access-related complications	3 (3%)	1 (5%)	1 (3.7%)	2 (1.5%)
Death (all causes)	2 (2%)	1 (5%)	0	3 (2.2%)
Stroke (all causes)	2 (2%)	0	1 (3.7%)	2 (1.5%)
Major bleeding	0	0	1 (3.7%)	14 (9.1%)
Pericardial/Pleural effusion	1 (1%)	3 (15%)	2 (7.4%)	16 (10.4%)

IQR, Interquartile range; TEE, Transesophageal echocardiography

* Values are given as the mean±SD

† Values are given as the median

## Notes:

***This paper should be cited as:*** Safavi-Naeini P, Razavi M, Saeed M, Rasekh A, Massumi A. A Review of the LARIAT Suture Delivery Device for Left Atrial Appendage Closure. J Teh Univ Heart Ctr 2015;10(2):69-73.
